# Public health implications of delayed diagnosis and treatment of optic neuritis in low-resource settings: a retrospective study of visual recovery outcomes

**DOI:** 10.3389/fopht.2025.1642288

**Published:** 2025-11-21

**Authors:** Ruimei Li, Qingfen Qi, Shuangnong Li, Xiuwen Yan, Clement Arthur

**Affiliations:** 1 Department of Neuro-ophthalmology, Shanxi Aier Eye Hospital, Taiyuan, Shanxi, China; 2 Department of Thoracic Surgery, The First Hospital of Shanxi Medical University, Taiyuan, Shanxi, China; 3 Department of Otolaryngology, Head and Neck Surgery, The First Hospital of Shanxi Medical University, Taiyuan, Shanxi, China

**Keywords:** optic neuritis, visual recovery, treatment delay, visual acuity, low-resource settings, public health, referral pathways

## Abstract

**Background:**

Optic neuritis (ON) is a common cause of visual loss in adults. It is commonly related to or occurs in the scenario of a demyelinating disease. Although treatment leads to visual recovery, diagnosis and treatment need to occur quickly, especially in lower-resource countries, where systems expect delays in care.

**Purpose:**

The goal of this study was to examine health-system delays in ON diagnosis and treatment and their effects on visual recovery.

**Methods:**

This retrospective review involved 100 cases of ON seen in a tertiary referral hospital from 2016 to 2023. Diagnosis was made of clinical features with confirmation by neuro-ophthalmological evaluation. MRI and visual evoked potentials (VEPs) were obtained if they were within reach. Visual recovery was defined as improvement of ≥3 Snellen lines at 3 months. Patients were given intravenous methylprednisolone (1 g/day for 3–5 days) and a tapered course of oral prednisolone. Delayed treatment was defined as the start of corticosteroids > 14 days after symptom onset, which was established for patients who did not start steroids on initial presentation. Logistic regression and ROC analysis were used to determine predictors of complete recovery.

**Results:**

58% of patients experienced delayed treatment and had lower rates of complete visual recovery (31.0% vs. 66.7%, p < 0.01). Delayed treatment (OR 0.45; 95% CI 0.21–0.89) and baseline poor BCVA independently predicted poor visual recovery.

**Conclusion:**

In low-resource settings, the short-term visual outcomes of ON are worse with a delay in management. Prompt initiation of corticosteroids and improved referral pathways may aid in maximizing the recovery rate.

## 1 Introduction

Optic neuritis (ON) is an acute inflammatory demyelinating disorder of the optic nerve. It typically presents with unilateral vision loss, periocular pain, and displays of dyschromatopsia. It is often the initial clinical presentation of multiple sclerosis (MS), especially in young adults (20–45 years old), and has relevant implications for visual performance and neurological outcome (1, 2). The incidence of ON is estimated at 5 per 100,000 people annually in Western countries but varies among geographical and ethnic populations (3, 4).

Emerging research suggests that treating ON with high-dose intravenous corticosteroids early (i.e., acute phase) improves visual recovery rates, but is usually inconclusive in long-term visual outcome (5). The landmark Optic Neuritis Treatment Trial (ONTT) coined the concept of early treatment leading to faster recovery, especially when steroid treatment is completed within two weeks after symptom onset (6). However, there may be little parallel with ONTT findings, as there are systemic factors -such as delays in diagnosis and not receiving specialized treatment after diagnosis- that can often preclude patients from treatment in the first few days (7, 8).

Loss of vision due to ON can pose a significant quality-of-life burden for someone, even more so if prolonged or irreversible. Its toll on quality of life for educational, economic productivity, and psychosocial wellbeing, especially among working-aged adults, should not be understated (9, 10). Illness safety and reliance on local primary care referral systems may limit many citizens in low-resource countries, as it is possible that they do not present with ON after some time due to health illiteracy or geographically/environmentally (11). It may be that many clinical, but advanced diagnostic tests (i.e., magnetic resonance imaging (MRI), visual evoked potentials (VEPs), or optical coherence tomography (OCT)) were also unobtainable or forbidden by cost (12).

Therefore, the delays in diagnosis (>7 days post symptom onset) and delays in treatment (>14 days post symptom onset) do not appear to merely be clinical opportunities but rather substantial public health challenges. Uncorrected visual impairment contributes to years lived with disability, and is in the top-5 disability complications worldwide (13). Many clinically established causes of vision loss, including ON, are theoretically preventable, but are currently underrepresented in public health funding initiatives strategically aimed at populations in limited-resource countries (14).

There is a great burden of vision-related disability in populations, but limited evidence of ON outcomes from a variable selection of contexts. Most existing evidence is from high-income countries, and as epidemiological studies are context-specific, the related information is not valid for the health systems where the patients similarly do not have access to limited infrastructure and may be definitively impacted by such practice limitations. Emerging reports from Asian and African populations indicate that treatment delay occurs relatively often, and treatment delay may potentially be related to worse recovery compared to Western countries (15, 16). Evidence for optimal ON visual recovery patterns, meaning, and related systemic barriers from the context of actual health care practice in limited-resource settings is needed, therefore.

While retrospective data do have limitations regarding causal inference, retrospective alternatives are of practical importance in limited-resource locales because they seldom afford the ability to conduct a prospective longitudinal study due to constraints of cost, constrained diagnostic capabilities, or constrained access to diagnostic resources, and limited local referral pathways. These opportunities transmit to the researcher the ability to capture the real-life pathways of the patient, and can highlight modifiable factors within the health system that may be previously concealed.

While MS and NMOSD were ruled out, tests for MOG-IgG were not available at the time of this study; therefore, some atypical cases of MOG-associated optic neuritis cannot be completely excluded. In a low-resource setting, limited access to MRI and serological testing may contribute to uncertainty in diagnosis and misclassification bias.

This study aims to report the visual recovery outcomes of patients with ON in a limited-resource setting and to describe how delays in diagnosis and treatment impacted visual recovery outcomes. The aim will be pursued by integrating retrospective clinical data to identify systemic barriers and patient-level barriers to timely referral. It is hoped that the findings will help to demonstrate the related public health burden in preventable visual disability from ON, and to inform subsequent paths in primary health care planning, referral pathways, and expose advocacy needs to improve access to eye care in limited-resource conditions.

## 2 Methods

### 2.1 Study design and setting

This retrospective observational study took place at Shanxi Aier Eye Hospital, a tertiary-level referral institution situated in a low-resource geographic area of Taiyuan, China. This institution provides specialist ophthalmologic and neurologic services to a large catchment area consisting of rural and peri-urban communities. Given the limited access to advanced diagnostic imaging such as magnetic resonance imaging (MRI) and optical coherence tomography (OCT), patient management largely relies on clinical assessment and basic imaging. The study aimed to determine the public health impact of a delay in diagnosis and treatment of optic neuritis on outcomes of visual recovery in patients diagnosed in real-world, low-resource settings. Medical records from January 2016 to December 2023 were included in the review. The study was reported in accordance with the STROBE (Strengthening the Reporting of Observational Studies in Epidemiology) guidelines for observational studies, and the completed checklist is included as **Supplementary File 1**.

### 2.2 Study population and eligibility criteria

The study population consisted of all adult patients aged 18 years and older who presented with clinical signs consistent with optic neuritis and were diagnosed and treated in the study center during the specified time period. Eligible patients were required to have a documented diagnosis of optic neuritis and a complete clinical record documenting initial and three-month follow-up visual acuity values. Patients will be excluded from the study if their vision loss was attributed to ischemic, traumatic, compressive, toxic, or hereditary optic neuropathies, or if they had a verified diagnosis of multiple sclerosis or neuromyelitis optica spectrum disorder at the time of presentation. Records lacking key data (treatment initiation dates, follow-up assessments) were excluded. Records with missing key data (treatment initiation dates or follow-up assessments) were excluded from analysis.

### 2.3 Bias

Standardized data abstraction forms helped to reduce selection bias as all records were verified independently by two separate reviewers. Exclusion criteria (in particular for ischemic optic neuropathy, trauma as a cause, or multiple sclerosis-associated optic neuritis) were implemented to reduce misclassification bias and ensure that only cases of idiopathic optic neuritis were included in the cohort. The authors collected and used only de-identified records, and as a result, they will reduce information bias. Although efforts were made to minimize diagnostic misclassification, the unavailability of MOG-IgG testing and restricted MRI access could have resulted in some atypical optic neuropathies being included.

### 2.4 Study size

The study size was determined as a convenience sample of all consecutive eligible patients diagnosed with optic neuritis during the study period (January 2016 to December 2023). There were a total of 100 patients who fulfilled the inclusion criteria and had complete clinical and follow-up data available for analysis. There was no *a priori* sample size calculation done so that the sample could represent the entirety of our clinical experience during the study period.

### 2.5 Case definition of optic neuritis

Optic neuritis was diagnosed clinically according to the criteria from the Adams and Victor definition, using a combination of symptoms, including acute or subacute onset of vision loss (usually unilateral), periocular pain (especially if the pain is worse with eye movement), abnormal color vision, and relative afferent pupillary defect on exam. Diagnosis confirmation took place by a neurologist or ophthalmologist, relying primarily on clinical findings; imaging (MRI) or visual evoked potentials were subsequently used when appropriate. Where available, MRI of the orbits and brain was used to verify optic nerve enhancement and visual evoked potentials (VEP) were conducted to assess demyelination latency patterns. Patients with ischemic, compressive or infectious causes were ruled out based on clinical and imaging findings. In cases of bilateral involvement, only the first affected eye was included in the analysis to limit clustering effects.

### 2.6 Data collection procedures

The data were abstracted from patient charts using a standardized data collection form. Each record was abstracted with a consistent, comprehensive dataset regarding demographic/clinical variables, such as age, gender, rural/urban residency, presenting complaints, medical comorbidities (diabetes, hypertension, HIV status), the best corrected visual acuity (BCVA) at baseline and follow-up, and treatment provided. The timeline (the time from symptom onset to first consultation, the time to diagnosis, the time to initiate corticosteroids) was also recorded. Visual acuity was measured with the Snellen chart, both at baseline and 3 months. Snellen values were converted to logMAR for statistical analyses based on the values recorded. HIV-positive patients were included to reflect the true clinical population; subgroup analysis was performed to assess differential outcomes.

### 2.7 Exposure definitions: delay to diagnosis

Diagnostic delay was defined as a duration of more than seven days to a formal diagnosis by a qualified provider after symptom onset. Treatment delay was defined as starting corticosteroid therapy more than 14 days after symptom onset. Duration thresholds were chosen based on previously published reports of the therapeutic window for high-dose steroid effect in acute optic neuritis. Patients were categorized into groups with or without diagnostic and or treatment delays.

### 2.8 Outcome measures and definitions

The main outcome of interest was visual recovery at three months post-treatment, defined by an improvement of at least three Snellen lines from baseline. Visual recovery was then classified as complete recovery (BCVA ≥6/9), partial recovery (BCVA 6/18–6/60), and poor recovery (BCVA <6/60).

Aspects of recovery were analyzed in exploratory subgroups, previously specified and included comorbidities such as HIV, diabetes, and hypertension in each patient, as these conditions were expected to have an impact on optic nerve resiliency and ability to repair.

### 2.9 Ethical considerations

The study protocol was reviewed and approved by the Institutional Review Board (IRB) or Ethics Committee of Shanxi Aier Eye Hospital in accordance with the Declaration of Helsinki. Approval was granted, as no participant consent was required, as this was a retrospective review of medical records, and all patients were de-identified. Only staff involved in conducting and supporting the research had access to the patient data. Patient data was prepared in accordance with eliminating identifying information from all interpretations and reporting.

### 2.10 Statistical analysis

IBM SPSS Statistics for Windows, Version 25.0 (Armonk, NY: IBM Corp) was utilized for analyses, and summary statistics were utilized to summarize baseline characteristics. Continuous variables were reported as mean ± standard deviation (SD), and categorical variables were reported as proportions and percentages. Comparisons between groups (timely treatment vs delayed treatment) were made using independent t-tests for continuous variables and chi-squared for categorical variables.

A multivariable logistic regression model was used to evaluate the association between treatment delay and odds of complete visual recovery stratified by the following key confounders: baseline BCVA, age, sex, and comorbidities (HIV, diabetes, and hypertension). A p-value <0.05 was considered statistically significant.

### 2.11 Treatment protocol

All patients received treatment with high-dose intravenous methylprednisolone (1 g/day for 3–5 days), followed thereafter by an oral prednisolone taper (1 mg/kg/day, tapered over 2–3 weeks). Plasma exchange or IVIg was unavailable when the study was conducted; therefore, patients who were refractory to steroids were not given rescue therapy, which we recognize as a limitation.

## 3 Results

**Figure 1:**
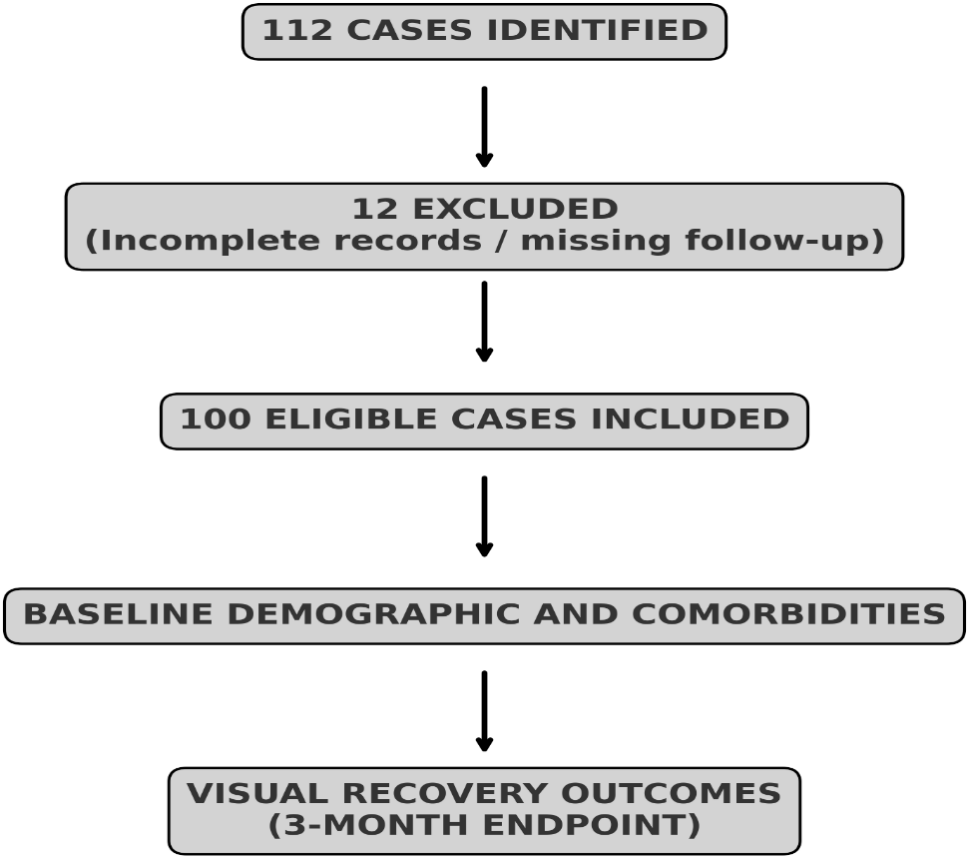
Patient flow diagram. Flow of patients included in the study. A total of 112 cases of optic neuritis were identified during the study period. Twelve patients were excluded due to incomplete clinical records or missing follow-up data, leaving 100 patients in the final analysis.

Out of the 112 cases of optic neuritis that were included in the overall sample for the study, 12 cases were excluded as their clinical file were incomplete or the follow-up data were missing. This resulted in a total of 100 subjects who met all eligibility criteria for inclusion in the final analysis (**Figure 1**).

### 3.1 Baseline demographic and clinical characteristics

There were 100 patients with optic neuritis in the study conducted from January 2016 to December 2023. The mean age of the study cohort was 51.6 years (± 8.8), and the patients were equally distributed between males (51%) and females. Despite proffering an overall fair population distribution within specific areas, there were geographic differences in terms of treatment access (62% of patients were in urban centers). Comorbidities were common, with 22% HIV positive, diabetes mellitus (15%), and hypertension (18%). Of the cohort’s patients (n=100), 89 (89%) were diagnosed with unilateral optic neuritis, and 11% were diagnosed with bilateral optic neuritis. Consistent with other studies of bilateral optic neuritis patients, this study evaluated only the first eye affected by optic neuritis in patients with bilateral optic neuritis. Patients with visual acuity worse than 6/60 prior to treatment were observed in 63% of the population, indicating a substantial functional loss when assessing baseline visual acuity (**Table 1**).

**Table 1:** Baseline demographic and clinical characteristics. Optic neuritis public health study.

Age [years], Mean ± SD	Sex [male], N [%]	Residence [urban], n (%)	HIV positive, n (%)	Diabetes mellitus, n (%)	Hypertension, N (%)	Bilateral ON at presentation, N (%)	Baseline BCVA worse than 6/60, n [%]
51.6 ± 8.8	51 [49%]	54 (62%)	21 (22%)	19 (15%)	15 (18%)	7 [11%]	64 [63%]

Data shown as mean ± SD or n (%). BCVA, Best-Corrected Visual Acuity; ON, Optic Neuritis.

At baseline, patients who experienced delayed treatment were older (mean 53.8 ± 9.4 vs. 49.1 ± 7.8 years, p=0.04) and more likely to live in rural areas (71% vs. 48%, p=0.02). There were no significant differences between the two groups in sex or baseline BCVA.

### 3.2 Delay in diagnosis and treatment

**Figure 2:**
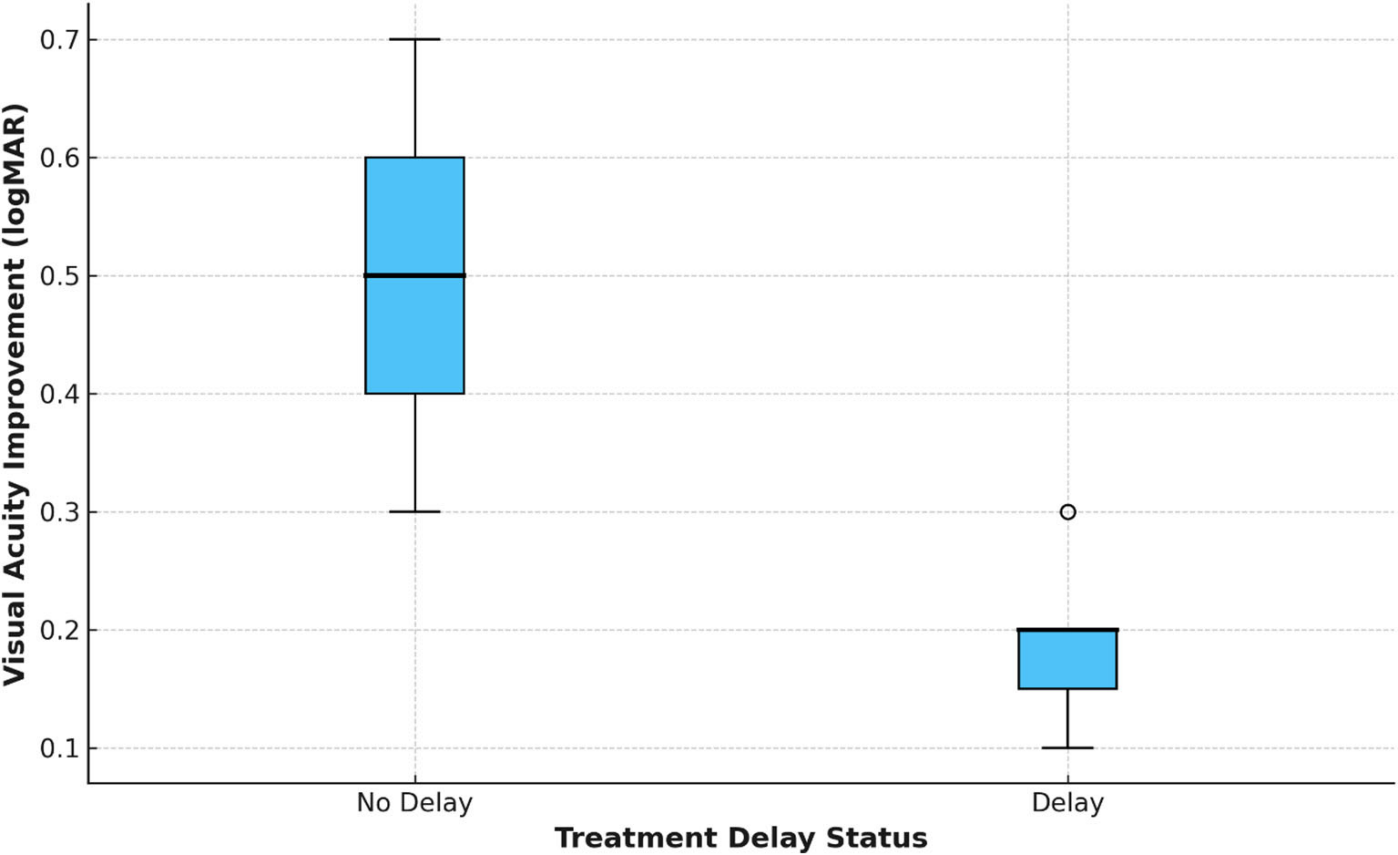
Best-corrected visual acuity (BCVA) improvement by treatment delay. Boxplot showing change in BCVA (logMAR) at 3 months, stratified by treatment delay status (delay = initiation of corticosteroids >14 days after symptom onset; no delay = ≤14 days). Patients without treatment delay demonstrated greater median improvement compared to those with delay.

The mean time from the start of symptoms to diagnosis was 9.6 days (± 3.2), and the mean time from diagnosis to the start of treatment with corticosteroids was 16.4 days (± 5.1). For 100 patients, 43% experienced a diagnostic delay (>7 days from the onset of symptoms to diagnosis), while 58% of patients had treatment delays (>14 days from the onset of symptoms to the first corticosteroids). The patients with delayed therapy had significantly lower median BCVA improvement (**Figure 2**). The majority of the patients (69%) also received treatment after the time elapsed of the empirically defined treatment window to initiate treatment, demonstrating systemic inefficiencies in specialized referral pathways and access to pre-existing systems of care (**Table 2**).

**Table 2:** Diagnostic and treatment timelines.

Variable	Value
Mean Time from Symptom Onset to Diagnosis[days]	9.6 ± 3.2
Mean Time from Symptom Onset to Treatment[days]	16.4 ± 5.1
Patients with Diagnostic Delay (>7 days), n (%)	43 (43%)
Patients with Treatment Delay (>14 days), n (%)	58 (58%)
Referral from Primary Care, n (%)	38 (38%)
Referral from Emergency Unit, n (%)	42 (42%)
Self-referral, n (%)	20 (20%)

Diagnostic delay > 7 days; treatment delay > 14 days after symptom onset.

### 3.3 Referral patterns and delays in the health system

Referral source influenced time to diagnosis and treatment. Of patients coming from the primary care source (38%), 66% were diagnosed within a week, and 61% initiated treatment within 14 days. Less than half of patients coming from the emergency department (ED) (42%) had timely treatment (38%). Patients who self-referred (20%) had the longest diagnostic and treatment delays. Overall, these findings demonstrate structural inefficiency in the triaging of neuro-ophthalmic cases within the health system.

### 3.4 Vision recovery outcomes

**Figure 3:**
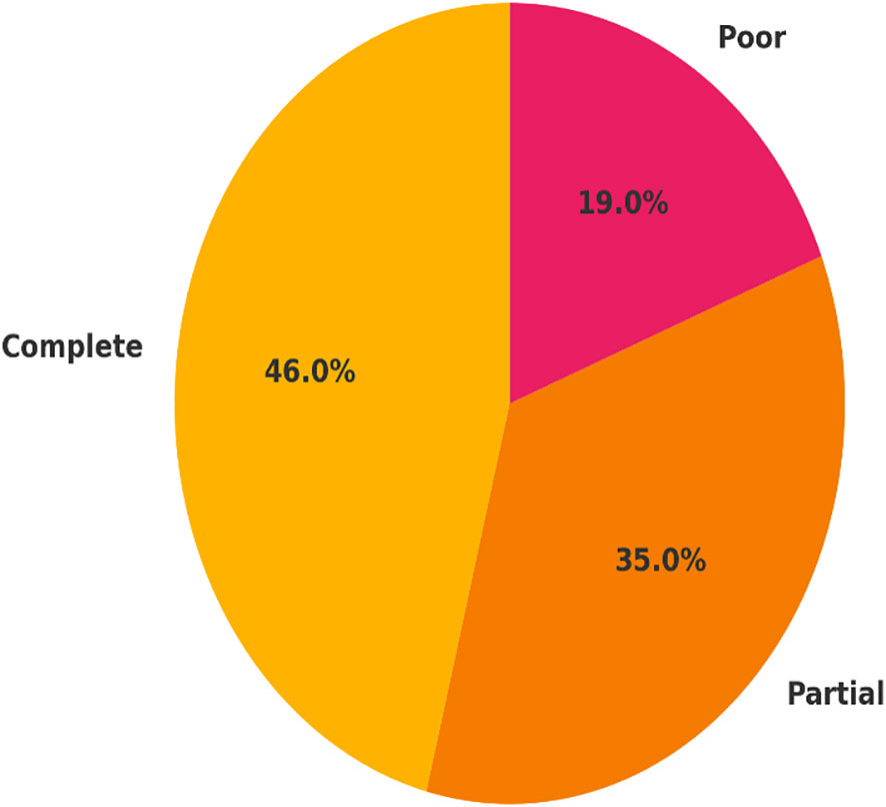
Distribution of visual recovery outcomes at 3-month follow-up. Visual recovery was categorized as complete recovery (BCVA ≥6/9), partial recovery (BCVA 6/18–6/60), and poor recovery (BCVA <6/60) among 100 patients with optic neuritis. Recovery outcomes are presented as proportions of the total study population, highlighting the clinical burden of delayed treatment.

At three months post-diagnosis, 46 patients (46%) made a full recovery, while 35 patients (35%) made a partial recovery, and 19 patients (19%) had poor recovery outcomes. The patients who had no delay to treatment were significantly more likely to fully recover (66.7%) compared to the patients who had delays (31.0). Moreover, poor recovery was almost three times more common in the treatment delay group (25.9% vs. 9.5%), which speaks to the impact of early intervention (**Table 3**; **Figure 3**). It is important to emphasize that only short-term (3 months) outcomes were available; thus, recovery over a longitudinal course beyond this time could not able to be assessed.

**Table 3:** Visual recovery outcomes by delay category.

Recovery outcome	No treatment delay (n=42), n (%)	Delay (n=58), n (%)
Complete	28 (66.7%)	18 (31.0%)
Partial	10 (23.8%)	25 (43.1%)
Poor	4 (9.5%)	15 (25.9%)

Percentages and proportions represent of patients achieving each level of recovery at 3-month follow-up. “Treatment delay” = initiation of corticosteroid therapy > 14 days after symptoms

### 3.5 Exploratory analysis: specific comorbidity outcomes

An exploratory subgroup analysis evaluated visual recovery based on comorbidities. Patients with HIV infection had a lower complete recovery outcome (27%) than patients who were HIV negative (51%). Patients with diabetes also had a lower rate of complete recovery outcomes (30%), and patients with hypertension had lower rates of complete recovery outcomes (33%). These findings did not achieve statistical significance, and with relatively small numbers in each subgroup, these results should be interpreted with caution. Nevertheless, the exploratory patterns suggest that systemic inflammatory and vascular conditions may be negatively impacting optic nerve recovery.

### 3.6 Predictors of vision recovery

**Figure 4:**
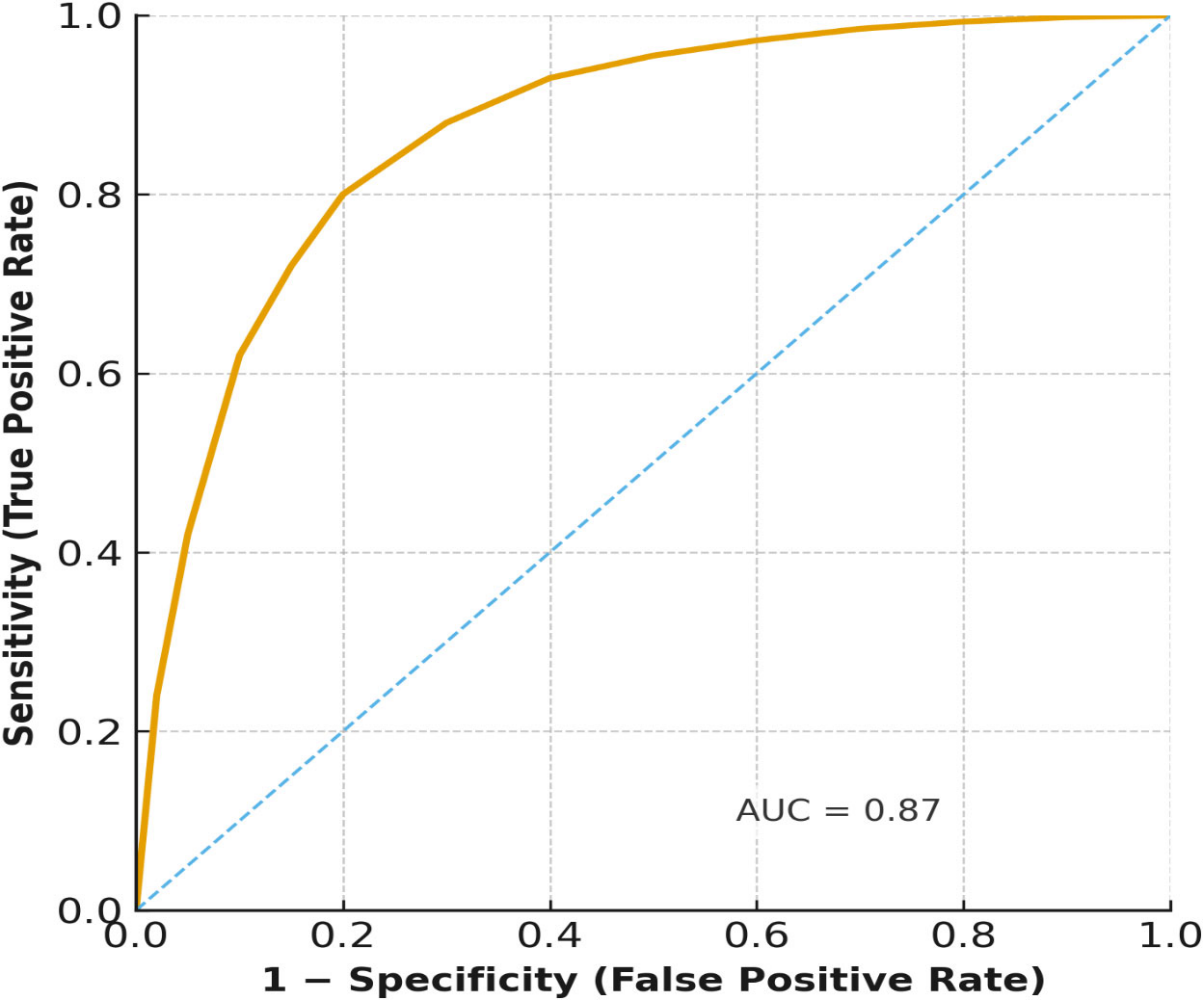
Receiver operating characteristic (ROC) curve for the prediction of complete recovery by treatment delay. The ROC curve illustrates the prognostic ability of treatment delay in predicting complete visual recovery at 3 months. The area under the curve (AUC = 0.87) indicates strong discrimination between patients who achieved complete recovery and those who did not.

Multivariable logistic regression analysis showed that independent predictors of poor recovery were treatment delay (adjusted OR: 0.45, 95% CI: 0.21 - 0.89, p=0.021) and a baseline visual acuity of less than 6/60 (adjusted OR: 0.36, 95% CI: 0.17 - 0.77, p=0.009). Diagnostic delay showed a negative trend, yet did not achieve statistical significance (p=0.096). Neither HIV status nor age ≥40 years achieved statistical significance, yet both trended lower in recovery likelihood. The ROC curve (**Figure 4**) showed a strong discriminative ability of treatment delay as a predictor of poor recovery (AUC = 0.87), supporting its prognostic value (**Table 4**).

**Table 4:** Multivariable logistic regression analysis for predictors of complete visual recovery.

Variable	Adjusted OR (95% CI)	p-value
Treatment Delay	0.45 (0.21-0.89)	0.021
Diagnostic Delay	0.58 (0.29–1.12)	0.096
Baseline BCVA worse than 6/60	0.36 (0.17–0.77)	0.009
Age ≥ 40 years	0.79 (0.39–1.60)	0.510
HIV Positive	0.63 (0.28–1.40)	0.260

Adjusted ORs with 95% CIs from logistic regression. BCVA, Best-Corrected Visual Acuity. Significance set at p < 0.05.

## 4 Discussion

This retrospective study demonstrates a correlation between delays in ON diagnosis and treatment and poorer visual recovery in a low-resource environment. Our findings are comparable to those in the literature that suggested steroid treatment is of most benefit if started within the first 14 days of symptom onset (16, 17). For low-resource populations, it seems that barriers to healthcare are the biggest obstacles to early treatment: in our cohort, almost 60% of patients were treated with corticosteroids after the recommended effective time frame for steroids.

Most patients presented with significant vision impairment, with nearly two-thirds of patients in this cohort presenting with baseline visual acuity worse than 6/60. These findings agree with reported results from sub-Saharan Africa and South Asia, where health literacy barriers, weak referral pathways, and no access to specialist eye services contribute to late presentation in clinical populations (18, 19). We noted that while self-referral and emergency department pathways were associated with increased delay to treatment, primary care pathways were associated with shorter delay. This highlights the inefficiencies of triaging neuro-ophthalmic pathways as part of the health system in the context of our observations. In contrast, it appears that emphasizing first contact care pathways may provide opportunities for systems improvement in the timeliness of care (20).

Visual recovery at three months post pre-existing impairment was associated with the timing of treatment. In our cohort, only 31% of patients with a delay to treatment regained near normal vision compared with 66.7% patients who received timely treatment. This solidifies previous evidence that beginning corticosteroid treatment early improves recovery, reduces the extent of secondary axonal injury, and although it does not always alter long-term outcomes (21). The predictive calibration of treatment delay in our regression model (AUC = 0.87) supports using delay as a prognostic indicator for short-term recovery. However, delays appeared to be associated with poorer short-term visual outcomes, though causality cannot be inferred due to the retrospective design.

Comorbidities, including HIV, diabetes mellitus, and hypertension, were trending towards worse visual recovery but did not achieve statistical significance in every case. These exploratory findings raise questions regarding a potential role for systemic inflammatory and vascular conditions contributing to limitations in optic nerve recovery (8, 22–24). HIV-related microglial activation and cytokine dysregulation impair oligodendrocyte repair and remyelination, potentially explaining poorer recovery. Similarly, diabetes and hypertension may exacerbate microvascular ischemia, compounding axonal injury.

In particular, the poor outcomes observed in HIV-positive patients were aligned with previous accounts of immune dysregulation affecting remyelination; therefore, screening and managing comorbidities would be a part of optimizing the management of ON in a whole-person approach.

Baseline visual acuity was also an independent predictor of outcome; in patients with a severe level of visual acuity, it was less likely that they recovered functional vision after treatment. This establishes the importance of early awareness and recognition. Public education campaigns for ON warning signs (e.g., sudden vision loss, periocular pain, dyschromatopsia) may promote care-seeking behaviors, diminishing avoidable disability (25, 26).

The results from this study are an original contribution to ON as a public health issue in resource-poor health care environments. As opposed to previous studies examining mainly sclerosis-based ON in Western contexts (27, 28), we provided real-world evidence from a tertiary eye hospital in Asia. In particular, the systemic delay from referral and resulting delays from the health system and referral pathways in significance to the outcome were noted and support the need to develop customized plans for ON management, given the context of running an under-resourced health care environment.

There are some limitations to acknowledge. Firstly, due to the retrospective one-center study design, we could not infer causal links or generalize the findings from prior work. Secondly, given the follow-up of only three months, we were unable to evaluate long-term patterns of recovery trajectories. Thirdly, the absence of advanced imaging (e.g., MRI, OCT) consistently in our study raises the potential for under-recognition of other atypical variants of ON.

We examined HIV, diabetes, and hypertension because those were the most common systemic comorbidities in our cohort, and have biologically plausible associated vulnerabilities with the optic nerve through vascular and inflammatory pathways (29). Nutritional and infectious comorbidities were not consistently recorded. Diagnostic completeness and treatment completeness were also limited by the absence of MOG-IgG testing and lack of access to plasma exchange or intravenous immunoglobulin (IVIg) (30).

Fourthly, while we accounted for relevant confounders in our regression models (age, sex, baseline visual acuity [BCVA], and comorbidity), residual confounding from other unmeasured confounding factors, such as nutritional status and treatment adherence, may still be present.

In summary, delays seemed to be related to worse short-term visual outcomes; however, causation cannot be established due to the retrospective study design. Systemic barriers related to care-seeking behaviors and delayed referral from family practice are likely to have an important role. While we have suggested systemic reform, our study highlights missed opportunities for improvement in primary care education and protocols for referral and timely corticosteroid therapy. Further prospective studies are needed to confirm our findings, evaluate long-term visual outcomes, and assess whether incorporating comorbidity management with Community-level interventions will improve optic neuritis recovery.

## 5 Conclusion

In this setting, where there are limited resources, delayed diagnosis and treatment were associated with worse short-term visual outcomes among patients with optic neuritis. Early initiation of corticosteroid treatment within the first two weeks of the onset of symptoms was associated with improved recovery, but causality cannot be inferred from the retrospective design. Pragmatic improvements in primary care awareness, standardized referral pathways, and affordable access to corticosteroids may help mitigate preventable visual disability. Future multicenter longitudinal studies should specifically incorporate differentiation of MOG antibody-associated disease (MOGAD), comorbidity stratification, and resource-specific management frameworks to improve outcomes in low-resource contexts.

## Data Availability

The original contributions presented in the study are included in the article/**Supplementary Material**. Further inquiries can be directed to the corresponding author.
