# Supplementary material for: Public health implications of delayed diagnosis and treatment of optic neuritis in low-resource settings: a retrospective study of visual recovery outcomes
Source: Front Ophthalmol (Lausanne). 2025 Nov 21;5:1642288. doi: 10.3389/fopht.2025.1642288 (PMC12678155; doi:10.3389/fopht.2025.1642288)
Supplement: Supplementary file 1 [file Table1.docx]

# STROBE Checklist for Observational Studies

This STROBE (Strengthening the Reporting of Observational Studies in Epidemiology) checklist has been completed for the manuscript: 'Public Health Implications of Delayed Diagnosis and Treatment of Optic Neuritis in Low-Resource Settings: A Retrospective Study of Visual Recovery Outcomes'. The checklist demonstrates how each STROBE item has been addressed in the manuscript.

| **Section / Item** | **Recommendation** | **Addressed in Manuscript** |
| --- | --- | --- |
| Title and Abstract | Indicate the study’s design with a commonly used term in the title or the abstract. Provide an informative and balanced summary of what was done and what was found. | Title specifies 'Retrospective Study'; Abstract provides objectives, methods, results, and conclusions. |
| Introduction: Background/Rationale | Explain the scientific background and rationale for the investigation being reported. | The introduction explains ON, ONTT evidence, and low-resource barriers. |
| Introduction: Objectives | State specific objectives, including any prespecified hypotheses. | The introduction final paragraph clearly states objectives (impact of diagnostic and treatment delay on recovery). |
| Methods: Study Design | Present key elements of the study design early in the paper. | Section 2.1 describes retrospective observational design. |
| Methods: Setting | Describe the setting, locations, and relevant dates, including periods of recruitment and follow-up. | Section 2.1 details Shanxi Aier Eye Hospital, 2016–2023. |
| Methods: Participants | Give the eligibility criteria, and the sources and methods of case ascertainment and selection. | Section 2.2 lists eligibility, exclusions (ischemic, traumatic, compressive, toxic, hereditary ON; MS/NMOSD). |
| Methods: Bias | Describe any efforts to address potential sources of bias. | Section 2.3: Standardized abstraction forms, double review, strict exclusion criteria, use of de-identified records. |
| Methods: Study Size | Explain how the study size was arrived at. | Section 2.4: All consecutive eligible patients (n=100); no a priori sample size calculation. |
| Methods: Variables | Clearly define all outcomes, exposures, predictors, potential confounders, and effect modifiers. Give diagnostic criteria, if applicable. | Section 2.5–2.6: Defines diagnostic delay (>7d), treatment delay (>14d), recovery outcomes, comorbidity subgroups. |
| Methods: Data Sources/Measurement | For each variable of interest, give sources of data and details of methods of assessment. | Section 2.6: Data abstracted from charts; Snellen converted to logMAR; standardized data form used. |
| Methods: Quantitative Variables | Explain how quantitative variables were handled in the analyses. | Section 2.6 and 2.10: BCVA categorized (≥6/9, 6/18–6/60, <6/60); regression adjusted for continuous covariates. |
| Methods: Statistical Methods | Describe all statistical methods, including those used to control for confounding. | Section 2.10: Logistic regression adjusted for baseline BCVA, age, sex, and comorbidities; p<0.05 significance level. |
| Results: Participants | Report number of individuals at each stage of study (e.g., eligibility, included, analysis). Give reasons for non-participation at each stage. | Results start: 112 identified, 12 excluded (missing data), 100 included in final analysis. |
| Results: Descriptive Data | Give characteristics of study participants (e.g., demographic, clinical, social). | Section 3.1: Age, sex, residence, comorbidities, unilateral/bilateral ON, baseline BCVA. |
| Results: Outcome Data | Report numbers of outcome events or summary measures over time. | Section 3.4: 46 complete, 35 partial, 19 poor recovery at 3 months. |
| Results: Main Results | Give unadjusted estimates and, if applicable, confounder-adjusted estimates and their precision (e.g., 95% CI). | Section 3.6: Logistic regression with adjusted ORs, 95% CIs, and p-values in Table 4. |
| Results: Other Analyses | Report other analyses done, e.g., subgroup analyses. | Section 3.5: Exploratory analyses of comorbidities (HIV, diabetes, hypertension). |
| Discussion: Key Results | Summarize key results with reference to study objectives. | The discussion first paragraph highlights the main findings (association of delays with poorer recovery). |
| Discussion: Limitations | Discuss study limitations, including sources of potential bias or imprecision. | Discussion: Retrospective design, single-center, 3-month follow-up, limited imaging, residual confounding. |
| Discussion: Interpretation | Give a cautious overall interpretation of results. | Discussion: Reframed as associations, 'missed opportunities,' contextualized within existing literature. |
| Discussion: Generalisability | Discuss the external validity of the study results. | Discussion emphasizes relevance to real-world, low-resource settings beyond Western ON studies. |
| Other Information: Funding | Give the source of funding and role of funders. | End of manuscript: No funding declared. |
